# PREDICT-juvenile-stroke: PRospective evaluation of a prediction score determining individual clinical outcome three months after ischemic stroke in young adults – a study protocol

**DOI:** 10.1186/s12883-022-03003-7

**Published:** 2023-01-04

**Authors:** Sonja Schönecker, Verena Hoffmann, Fady Albashiti, Reinhard Thasler, Marlien Hagedorn, Marie-Luise Louiset, Anna Kopczak, Jennifer Rösler, Enayatullah Baki, Silke Wunderlich, Florian Kohlmayer, Klaus Kuhn, Martin Boeker, Johannes Tünnerhoff, Sven Poli, Ulf Ziemann, Oliver Kohlbacher, Katharina Althaus, Susanne Müller, Albert Ludolph, Hans A. Kestler, Ulrich Mansmann, Marianne Dieterich, Lars Kellert

**Affiliations:** 1 Department of Neurology, Ludwig-Maximilians-Universität München, Marchioninistr. 15, D-81377 Munich, Germany; 2 Institute for Medical Informatics, Biometry, and Epidemiology, Ludwig-Maximilians-Universität München, Munich, Germany; 3 Center for Medical Data Integration and Analysis, Ludwig-Maximilians-Universität München, Munich, Germany; 4 Institute of Laboratory Medicine, Ludwig-Maximilians-Universität München, Munich, Germany; 5 Institute for Stroke and Dementia Research (ISD), Ludwig-Maximilians-Universität München, Munich, Germany; 6 Department of Neurology, School of Medicine, Klinikum rechts der Isar, Technical University of Munich, Munich, Germany; 7 Institute of Medical Informatics, Statistics and Epidemiology, University Hospital rechts der Isar of the Technical University Munich, Munich, Germany; 8 Department of Neurology & Stroke, Eberhard Karls University Tübingen, Tübingen, Germany; 9 Hertie Institute for Clinical Brain Research, Eberhard Karls University Tübingen, Tübingen, Germany; 10 Department of Computer Science, Center for Bioinformatics and Quantitative Biology Center, Eberhard-Karls-University Tübingen, Tübingen, Germany; 11 Max Planck Institute for Developmental Biology, Tübingen, Germany; 12 Department of Neurology, University of Ulm, Ulm, Germany; 13 Intitute of Medical Systems Biology, University of Ulm, Ulm, Germany; 14 Pettenkofer School for Public Health, Munich, Germany; 15 German Center for Neurodegenerative Diseases (DZNE), Ludwig-Maximilians-Universität München, Munich, Germany; 16 German Center for Vertigo and Balance Disorders, Ludwig-Maximilians-Universität München, Munich, Germany; 17 Munich Cluster for Systems Neurology (SyNergy), Munich, Germany

**Keywords:** Validation, Prediction score, Three months outcome, Juvenile stroke

## Abstract

**Background:**

Although of high individual and socioeconomic relevance, a reliable prediction model for the prognosis of juvenile stroke (18–55 years) is missing. Therefore, the study presented in this protocol aims to prospectively validate the discriminatory power of a prediction score for the 3 months functional outcome after juvenile stroke or transient ischemic attack (TIA) that has been derived from an independent retrospective study using standard clinical workup data.

**Methods:**

PREDICT-Juvenile-Stroke is a multi-centre (*n* = 4) prospective observational cohort study collecting standard clinical workup data and data on treatment success at 3 months after acute ischemic stroke or TIA that aims to validate a new prediction score for juvenile stroke. The prediction score has been developed upon single center retrospective analysis of 340 juvenile stroke patients. The score determines the patient’s individual probability for treatment success defined by a modified Rankin Scale (mRS) 0–2 or return to pre-stroke baseline mRS 3 months after stroke or TIA. This probability will be compared to the observed clinical outcome at 3 months using the area under the receiver operating characteristic curve. The primary endpoint is to validate the clinical potential of the new prediction score for a favourable outcome 3 months after juvenile stroke or TIA. Secondary outcomes are to determine to what extent predictive factors in juvenile stroke or TIA patients differ from those in older patients and to determine the predictive accuracy of the juvenile stroke prediction score on other clinical and paraclinical endpoints. A minimum of 430 juvenile patients (< 55 years) with acute ischemic stroke or TIA, and the same number of older patients will be enrolled for the prospective validation study.

**Discussion:**

The juvenile stroke prediction score has the potential to enable personalisation of counselling, provision of appropriate information regarding the prognosis and identification of patients who benefit from specific treatments.

**Trial registration:**

The study has been registered at https://drks.de on March 31, 2022 (DRKS00024407).

## Background

With an annual mortality rate of about 6.55 million and the loss of 143 million disability-adjusted life-years, stroke is ranked the second leading cause of death worldwide and is one of the most frequent causes of disability in adults [1]. While the incidence of stroke increases with age, about 15% of strokes occur in persons younger than 55 years [2], i.e. juvenile stroke, and of particular note is that the incidence of juvenile stroke increased up to 40% over the last decades [3]. Underlying aetiologies of stroke differ between younger and older patients and in juvenile stroke rare and cryptogenic causes are frequent with a range of 50% [4]. Due to the young age and longer life expectancy in juvenile stroke, the prognosis and therefore counselling differs from that in older patients. The long-term medical, psychosocial and socioeconomic consequences are particularly severe in this young age group [5]. Therefore, the identification of risk factors and the development and validation of a reliable prediction score for the outcome after juvenile stroke or transient ischemic attack (TIA) are urgently needed. To date there is no study that prospectively validates a model that predicts functional outcome after juvenile stroke. The PREDICT-Juvenile-Stroke study (https://drks.de; DRKS00024407) presented in this protocol aims to fill this gap by prospectively validating a juvenile stroke prediction score for the 3 months functional outcome that has been derived from an independent retrospective cohort study, using a combined set of clinical and paraclinical data.

## Methods/design

### Design

**Fig. 1:**
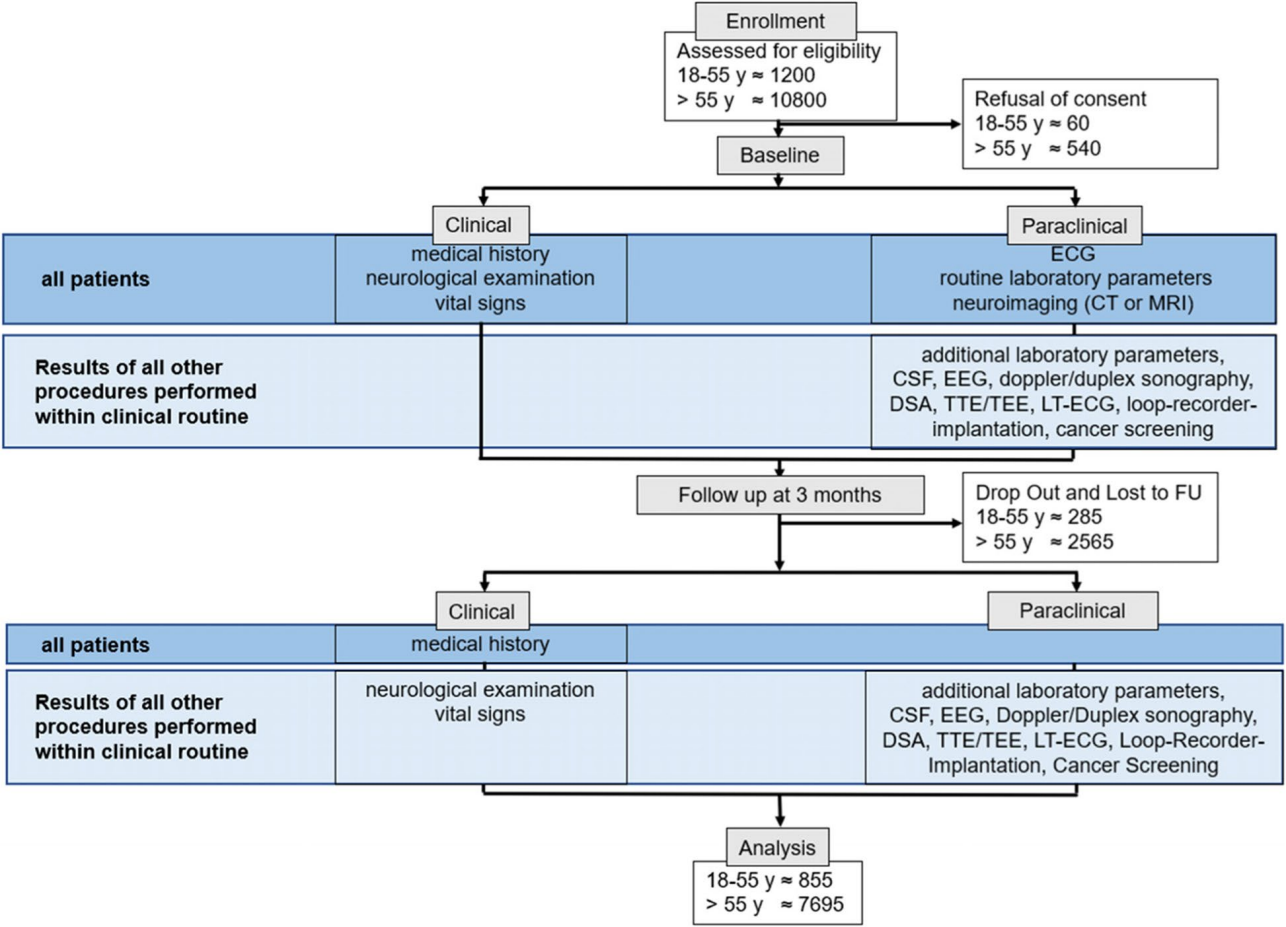
Graphical study design

The PREDICT-Juvenile-Stroke study presented in this protocol is a multi-centre, prospective observational cohort study on juvenile stroke or TIA. Patients are enrolled at the participating study sites at the university hospitals of the Ludwig-Maximilians University Munich, the Technical University Munich, the Eberhard Karls University Tübingen and the Ulm University Medical Center in Germany. The study records clinical routine data of newly diagnosed ischemic stroke or TIA patients according to current diagnostic criteria [6, 7] and involves phenotypic characterization over 3months including brain imaging as well as prospective biosample collection (Fig. 1). During the in-hospital stay routinely collected data including demographic data, clinical parameters as well as paraclinical data from examinations necessary within the framework of clinical workup (e.g., CT, MRI, doppler/duplex sonography, transthoracic echocardiography, veinpuncture, lumbar puncture) are recorded. Standardized clinical follow-up is assessed 3 months after stroke either as a routinely arranged outpatient visit or by a structured telephone interview. In each of the centres, the study uses a specific uniform IT-infrastructure (already established within the DIFUTURE consortium [8], supported by the BMBF grants 01ZZ1804A, 01ZZ1804B, 01ZZ1804C, 01ZZ1804D and 01ZZ1804I) in which clinical process data are transformed into structured data usable in clinical research. The recruitment phase lasts 3 years (Table 1). The derived data will be used to validate a juvenile stroke prediction score, which has been developed based on monocentric retrospective data of 340 juvenile stroke and TIA patients who were hospitalized at the stroke unit of Ludwig-Maximilians University Munich between Jan 01, 2011, and Mar 31, 2020. The score is based on predictor variables that are divided into 4 categories: preadmission factors; clinical, imaging and laboratory findings at admission; results of diagnostic investigations during the in-hospital stay and treatment given at admission or discharge; and is of high predictive value (area under the curve (AUC) 96.4%). As the binary parameter “ischemic stroke vs. TIA” did not improve outcome prediction, both stroke as well as TIA patients are enrolled in the presented study. Furthermore, prospective data will be used to determine to what extent variables included in the score differ from predictive factors in older stroke or TIA patients and determine the additive value of the new juvenile stroke prediction score compared to established age-independent prediction models. Another aim of the PREDICT-Juvenile-Stroke study is the assessment and prediction of quality of life in patients with juvenile stroke and older stroke patients. To characterize disease effect on quality of life, patient-reported outcome measures are assessed during the in-hospital stay and at the 3 months follow-up. These data will give new insights of modern therapies in stroke with respect to their long-term impact on quality of life.

**Table 1:** Timeframe of the study

Study Phase	Tasks	2023				2024				2025				2026			
		quarter (proposed trial duration)															
		Q1	Q2	Q3	Q4	Q1	Q2	Q3	Q4	Q1	Q2	Q3	Q4	Q1	Q2	Q3	Q4
Execution	Screening and inclusion period																
	3 months follow-up																
	possible extended Follow-Up																
Analysis	Final data analysis																
	preparataion of manuscript																

### Patient population – inclusion and exclusion criteria

On admission to the hospital, patients with acute ischemic stroke or TIA are screened for participation in the study.

### Inclusion criteria

- Juvenile stroke patients aged 18–55 years and older stroke patients > 55 years, both patients with first-ever strokes as well as with recurrent events can be enrolled
- Written informed consent by the patient or their legal representatives obtained at the latest prior to the 3 months follow-up

### Exclusion criteria

- The patient is directly involved in the conduct of the protocol
- Refusal of consent

Written informed consent can be obtained latest prior to the 3 months follow-up. As all information are collected as part of clinical routine, patients can be enrolled, and information be recorded even if patients are incapable of providing consent and no legal representative is available. If patients are lost to follow-up or die before informed consent can be obtained, the recorded data are kept within the study database in a de-identified manner.

### Treatment or intervention

Treatment decisions are entirely the responsibility of the treating physician and must follow state of the art best-practice rules.

### Primary outcomes

The primary objective is to prospectively validate the clinical potential of a juvenile stroke prediction score. Treatment success at 3 months is defined as mRS (modified Rankin Scale) [9] 0–2 or return to baseline pre-stroke mRS.

### Secondary outcomes

Secondary outcomes are to determine to what extent predictive factors in juvenile stroke or TIA patients differ from those in older patients, to correlate the juvenile stroke prediction score with secondary clinical and para-clinical endpoints (e.g. recurrent ischemic stroke or TIA or haemorrhagic stroke) and to determine its predictive accuracy with respect to these secondary endpoints.

A non-clinical objective is also to assess the properties and functionality of data flows implemented with the IT-infrastructure.

Demographic and clinical data to be collected (Fig. 1):

The following parameters are included in the juvenile stroke prediction model and are therefore collected to validate the model:

- Preadmission factors (age, CHA_2_DS_2_-VASc-Score)
- Clinical, imaging and laboratory findings at admission (National Institutes of Health Stroke Scale (NIHSS), Alberta stroke programme early CT score (ASPECTS), glucose level))
- Results of diagnostic investigations during the in-hospital stay (mean intima-media thickness on ultrasonography, underlying aetiology)
- treatment given at admission or discharge (intravenous thrombolysis, reperfusion success measured by the modified Thrombolysis in Cerebral Infarction (mTICI) score, antithrombotic therapy given at discharge)

Additionally, the following data are collected for further research during the in-hospital stay:

- Demographic data (gender, ethnicity)
- Medical/surgical history (pre-existing illnesses/previous operations, cardiovascular risk factors, signs and symptoms, onset of symptoms, premorbid mRS)
- Relevant concomitant treatment (type of therapy, name of medication, daily dosage and duration for such use)
- family history (positive family history defined as cardiovacular event in a first-degree relative < 65 years)
- Neurological examination (NIHSS after 24 hours as well as at discharge)
- Vital signs (blood pressure, heart rate, oxygen saturation, blood glucose)
- Patient-reported outcome measures (clinical global impression)

The following parameters are collected 3 months after index stroke:

- Medical/surgical history (new illnesses, operations that have taken place during the interval, cardiovascular risk factors, current signs and symptoms, mRS)
- Relevant medication (type of therapy, name of medication, daily dosage and duration for such use)
- Patient-reported outcome measures (clinical global impression, EuroQol – Visual analogue scale)
- Questionnaires on quality of life and depression (Beck-Depression-Inventory, EuroQol 5 dimensions with 5 response levels)
- Cognitive assessment (Montreal Cognitive Assessment (MoCA))
- Neurological examination (NIHSS)
- Vital signs

Paraclinical parameters to be documented:

- Electrocardiogram
- Laboratory analyses
- Neuroimaging (cerebral CT/CT angiography, CT perfusion, MRI, MR angiography)
- Digital subtraction angiography (DSA)
- Additional imaging modalities (e.g., X-ray, positron emission tomography)
- Doppler/duplex sonography
- Electroencephalography
- Transthoracic echocardiography/ transoesophageal echocardiography
- Long-term electrocardiogram
- Cancer screening (e.g. X-ray, CT-thorax/abdomen, gynecologic/dermatologic examination)
- Loop-recorder implantation
- Questionnaires
- CSF analysis
- Additional laboratory analyses

There is a variety of types of data and parameters which are documented if the respective examinations are necessary within the framework of clinical workup. For all procedures we assess if it is performed and if so, document the results.

### Biosamples

Patients can optionally agree to an additional collection of biomaterial (blood/CSF) at baseline as well as, if possible, at the 3 months follow-up visit as part of the routine diagnostic procedure (e.g. veinpuncture, lumbar puncture). One part of biosamples obtained from the patients is used for routine analysis. Here, clinical-chemical parameters, hematological examinations, functional characteristics and coagulation tests are performed. The other part is biobanked for biomarker studies, primary cells are isolated and cryopreserved. As a secondary objective we will analyse the predictive relevance of established biomarkers in the field like copeptin, serum neurofilament light chain, growth differentiation factor 15, S100, N-terminal pro-B-type natriuretic peptide, atrial natriuretic peptide and fatty acid-binding protein on treatment success and the 3 months functional outcome.

Further possible biomarkers are genome, epigenome, metabolome, proteome and transcriptome markers as well as parameters of morphological analytics and in vitro studies on isolated cells. Methods for analyzing proteins are among others ELISA, mass spectrometry and 2-dimensional gel electrophoresis. Methods for the analysis of RNA and DNA are for example polymerase chain reaction, microarray and sequencing. The metabolome and lipidome can be assessed with mass spectrometry.

### Data monitoring body

As all information are collected within the framework of clinical routine, no central data monitoring body is installed. Staff in the respective Neurology Departments is regularly trained and certified in the use of standardized scales and scores (e.g. National Institutes of Health Stroke Scale i.e. NIHSS and modified Rankin Scale, i.e. mRS). Data sets will automatedly be checked for plausibility before the final analysis. Federated privacy-preserving record-linkage is used to control for duplicates in the differing databases.

### Sample size estimates

The primary endpoint will be analysed by determining the receiver operating characteristic curve (ROC) and its AUC. We want to reject the null hypothesis: AUC ≤ 0.7. Assuming a true AUC of 0.8 and a treatment success rate of 0.7 a total of 344 patients is needed (power 90%, level of significance 5%). To compensate for possible missing data a minimum of 430 juvenile stroke/TIA patients aged 18 to 55 years will be included in the study (estimated 25% missing rate). To assess differences in predictive factors between juvenile and older stroke/TIA patients, the same number of older patients above 55 years will be included. However, the calculated number only provides the minimum number of patients for validation of the prediction score. We will include all eligible patients to increase power and facilitate further research. As about 100 juvenile acute stroke/TIA patients are treated annually in each of the participating stroke units, we expect to screen about 1200 juvenile stroke/TIA patients for inclusion in the study during the recruitment phase of 3 years.

### Statistical analyses

In order to test the null hypothesis, we use the 95% confidence interval of the AUC. The null hypothesis will be rejected if the value of 0.7 is not contained in the corresponding 95% confidence interval.

Additionally, the calibration curve and the Brier score will be calculated to assess calibration. We will also analyse the predictive value of the score with respect to alternative clinical and paraclinical outcome variables using the AUC of the ROC, calibration curves and the Brier score. According to data type, distributions and proportions will be compared and regression models will be fitted to identify age-related differences.

## Discussion

The juvenile stroke prediction score has the potential to enable personalisation of counselling, provision of appropriate information regarding the prognosis and identification of patients who benefit from specific treatments and rehabilitative strategies. As the score allows discrimination of prognostic strata of patients, it may also be of high value in designing research studies on secondary preventive as well as rehabilitative treatments following juvenile stroke and may therefore improve patient care as well as stroke research. Furthermore, the collected data will enable activities to identify new biomarkers, validate findings of comparable studies, development of assays for patient stratification and response to existing treatments. The data also contributes to meta-analyses, which aim to validate specific biomarker hypothesis. Besides, the study aims to demonstrate that clinical research can be performed in a more parsimonious way using the full scope of data being documented in clinical routine. The usage of a uniform IT-infrastructure (DIFUTURE [8]) that enables to collect harmonized data within the existing clinical IT, thereby avoiding parallel routine and study specific structures, represents a key strength of the study. This enables the inclusion of a high number of patients, contributes to increase quality and standardisation of routine clinical data and allows to simultaneously validate the discriminatory power of the prediction score prospectively in independent cohorts at different study centres. Another advantage of the study is the added value of a standardised follow-up across 4 centres.

Furthermore, the inclusion process represents a methodological strength of the study. In several previous studies on stroke the consent procedure excluded patients not capable of providing consent within a certain timeframe [10], which may have led to relevant selection bias by not providing consecutive data and excluding severely affected patients. In contrast, in PREDICT-Juvenile-Stroke the inclusion process ensures consecutive enrolment of patients thereby avoiding selection bias as well as allowing for a high inclusion rate independent of the patient’s capacity to provide consent.

### Summary and conclusion

The current study is the first that aims to prospectively validate the clinical potential of a prediction score for the 3 months functional outcome after juvenile stroke. This score has the potential to enable personalized patient care, provision of appropriate information regarding the prognosis and the identification of patients who benefit from a specific treatment.

## Data Availability

The datasets generated and/or analysed during the presented study are not publicly available due to protected health information but are available from the corresponding author upon reasonable request.

## Abbreviations

AUC: Area under the curve
mRS: Modified Rankin Scale
ROC: Receiver operating characteristic
TIA: Transient ischemic attack
